# Genome‐wide association study in Finnish twins highlights the connection between nicotine addiction and neurotrophin signaling pathway

**DOI:** 10.1111/adb.12618

**Published:** 2018-03-13

**Authors:** Jenni Hällfors, Teemu Palviainen, Ida Surakka, Richa Gupta, Jadwiga Buchwald, Anu Raevuori, Samuli Ripatti, Tellervo Korhonen, Pekka Jousilahti, Pamela A.F. Madden, Jaakko Kaprio, Anu Loukola

**Affiliations:** ^1^ Institute for Molecular Medicine Finland (FIMM) University of Helsinki Finland; ^2^ Department of Public Health University of Helsinki Finland; ^3^ Department of Adolescent Psychiatry Helsinki University Central Hospital Finland; ^4^ Wellcome Trust Sanger Institute UK; ^5^ Institute of Public Health and Clinical Nutrition University of Eastern Finland Finland; ^6^ National Institute for Health and Welfare Finland; ^7^ Department of Psychiatry Washington University School of Medicine Saint Louis MO USA

**Keywords:** Finnish population‐based imputation reference panel, genome‐wide association analysis, neurotrophin signaling pathway, nicotine addiction, nicotine withdrawal, smoking behavior, smoking quantity

## Abstract

The heritability of nicotine dependence based on family studies is substantial. Nevertheless, knowledge of the underlying genetic architecture remains meager. Our aim was to identify novel genetic variants responsible for interindividual differences in smoking behavior. We performed a genome‐wide association study on 1715 ever smokers ascertained from the population‐based Finnish Twin Cohort enriched for heavy smoking. Data imputation used the 1000 Genomes Phase I reference panel together with a whole genome sequence‐based Finnish reference panel. We analyzed three measures of nicotine addiction—smoking quantity, nicotine dependence and nicotine withdrawal. We annotated all genome‐wide significant SNPs for their functional potential. First, we detected genome‐wide significant association on 16p12 with smoking quantity (P = 8.5 × 10^−9^), near CLEC19A. The lead‐SNP stands 22 kb from a binding site for NF‐κB transcription factors, which play a role in the neurotrophin signaling pathway. However, the signal was not replicated in an independent Finnish population‐based sample, FINRISK (n = 6763). Second, nicotine withdrawal showed association on 2q21 in an intron of TMEM163 (P = 2.1 × 10^−9^), and on 11p15 (P = 6.6 × 10^−8^) in an intron of AP2A2, and P = 4.2 × 10^−7^ for a missense variant in MUC6, both involved in the neurotrophin signaling pathway). Third, association was detected on 3p22.3 for maximum number of cigarettes smoked per day (P = 3.1 × 10^−8^) near STAC. Associating CLEC19A and TMEM163 SNPs were annotated to influence gene expression or methylation. The neurotrophin signaling pathway has previously been associated with smoking behavior. Our findings further support the role in nicotine addiction.

## Introduction

Smoking is a major risk factor for non‐communicable diseases, with the largest public health burden due to chronic obstructive pulmonary disease, cancers and cardiovascular diseases (USDHHS 2014). Thus, tobacco use constitutes the most common cause of mortality, with more than 5 million preventable deaths resulting from direct tobacco use each year (WHO 2015).

For the majority of smokers, persistent tobacco use is motivated by nicotine dependence (ND) (Moss *et al*. 2012). Nicotine binds to nicotinic acetylcholine receptors (nAChRs) in the brain. Stimulation of nAChRs induces the release of various neurotransmitters, such as dopamine, which has a key role in drug‐induced reward by creating the perceptions of pleasure and reward (Nestler 2005). Repeated exposure to nicotine leads to neuroadaptation (Wang & Sun 2005), during which the number of nAChRs increases, plausibly due to desensitization of the receptors (Govind *et al*. 2009). Desensitization is suggested to mediate tolerance and dependence (Dani & Harris 2005). The symptoms of craving and withdrawal emerge during abstinence, when the desensitized receptors again become responsive (Dani & Harris 2005). Smoking and other forms of nicotine use alleviate these symptoms, as nicotine re‐binds to the receptors.

When attempting cessation, nicotine withdrawal (NW) symptoms cause powerful stimuli to sustain smoking (Le Moal & Koob 2007). These symptoms are strong predictors of relapse, specifically during the first week of a quit attempt (Ashare *et al*. 2013). The positive reinforcement induced by the dopamine system combined with the objective of avoiding NW symptoms underlies the pharmacological and physiological aspects of ND, while social and psychological factors add multi‐dimensionality (Benowitz 2010).

Family and twin studies have suggested high (40–75 percent) heritability for ND (Rose *et al*. 2009). Genome wide association study (GWAS) meta‐analyses have robustly reported a smoking behavior locus on 15q24–25 harboring genes encoding nAChR subunits α5 (*CHRNA5*), α3 (*CHRNA3*) and β4 (*CHRNB4*). Associations have been reported for numerous smoking‐related traits (Lassi *et al*. 2016). A functional variant D398N has been identified in *CHRNA5* (Bierut *et al*. 2008); however, alleles at this locus explain less than 1 percent of the variance in amount smoked (Thorgeirsson *et al*. 2008), and about 4–5 percent of the variance in cotinine levels (Keskitalo *et al*. 2009; Munafo *et al*. 2012). Still, the underlying genetic architecture of ND remains poorly understood.

Due to the design of the genotyping arrays and quality control settings, most variants highlighted in the previously mentioned large‐scale meta‐analyses have been common (minor allele frequency (MAF) >5 percent). Evidently, common variants of at least moderate effect only explain a fraction of the estimated heritability. Utilizing population‐specific imputation reference panels obtained from large‐scale whole‐genome/exome sequencing studies is shown to increase the imputation accuracy of low frequency (MAF 1–5 percent) and rare variants (MAF <1 percent) (Surakka *et al*. 2016). This may offer a means to shorten the gap between family‐based and measured genotype‐based genetic variance, i.e. for locating and patching up the hidden heritability.

To investigate the impact of common and low‐frequency variants on three distinct measures of nicotine addiction—smoking quantity, ND and NW—we conducted a GWAS in 1715 participants from the Finnish twin family study. Our genotype data were imputed using both the 1000 Genomes Phase I reference panel (1000 Genomes Project Consortium *et al*. 2012) and an all‐Finnish reference panel from the Sequencing Initiative Suomi (SISu) (sisuproject.fi). In this study, we identified novel loci accounting for interindividual differences in NW and smoking quantity.

## Materials and Methods

### Participants

The sample collection has been previously described in detail (Broms *et al*. 2007). Briefly, the study sample was drawn from the population‐based Finnish Twin Cohort Study, which consists altogether of 35 834 adult twins born in 1938–1957. Twin pairs concordant for ever‐smoking were identified and recruited along with their family members (mainly siblings) for the Nicotine Addiction Genetics (NAG) Finland study. Priority was given to heavier smokers. The data collection took place in 2001–2005. Participants were assessed by DNA sample collection and a structured diagnostic psychiatric interview resulting in detailed phenotypic information on multiple smoking behavior traits. The study has been approved by the Ethics Committee of the Hospital District of Helsinki and Uusimaa in 2001 and 2016, and by the IRB of Washington University, St. Louis, MO.

The study sample consisted of 1715 individuals with both phenotype and genotype data available (58 percent males, mean age 55 years, all smoked at least 100 cigarettes during lifetime) from 739 families, including 796 dizygotic (DZ) twins from 398 DZ twin pairs, 182 singletons (DZ twins without the co‐twin), 138 singletons (monozygotic (MZ) co‐twins randomly selected from a MZ twin pair), 49 twin participants with unconfirmed zygosity (due to a lack of DNA sample from the co‐twin) and 550 other family members (mainly siblings of the twins).

### Phenotypes

Participants were interviewed by trained interviewers (non‐psychiatrists) using the diagnostic Semi‐Structured Assessment for the Genetics of Alcoholism (Bucholz *et al*. 1994), modified for use in the Finnish population. The interview included a section on nicotine use and ND, based on the Composite International Diagnostic Interview (Cottler *et al*. 1991). In this study, we assessed the amount smoked, defined as self‐reported cigarettes per day (CPD) during the period of heaviest smoking, and the largest number of cigarettes smoked during a 24‐hour period (MaxCigs24). During the interview, CPD was assessed as a categorical variable of eight categories (1–2, 3–5, 6–10, 11–15, 16–19, 20–25, 26–39, ≥40 CPD during the period of heaviest smoking); in the analyses, we used class means of each category. MaxCigs24 was assessed and analyzed as a quantitative variable. We also assessed ND and NW based on the Diagnostic and Statistical Manual of Mental Disorders (DSM), 4^th^ edition (DSM‐IV) criteria (American Psychiatric Association 1994), both as binary diagnosis traits and as quantitative symptom counts. DSM‐IV ND diagnosis requires the presence of at least three out of seven criteria (during a 12‐month period). The DSM‐IV NW diagnosis requires the presence of at least four out of eight symptoms within 24 hours after an abrupt cessation of nicotine use or a deduction in the amount of nicotine use. DSM‐IV criteria for both phenotypes have been described in Supporting Information Document S1. Table 1 describes the basic statistics of the data.

**Table 1:** Descriptive statistics of the sample and phenotypes.

	Mean (min‐max; SD) or % for binary variables	*n* (with genotype information)
% males	57.8%	1715
Age	55.2 (30–91; 7.2)	1715
CPD	18.9 (1.5–45; 10.2)	1715
MaxCigs24	28.7 (1–80; 13.9)	1711
DSM‐IV NW symptoms	2.3 (0–8; 2.1)	1703
DSM‐IV‐NW diagnosis	31.7%	540 (cases), 1163 (controls)
DSM‐IV ND symptoms	2.9 (0–7; 1.7)	1715
DSM‐IV‐ND diagnosis	50.9%	873 (cases), 842 (controls)

### Genotyping and imputation

Genotyping was performed with the Human670‐QuadCustom Illumina BeadChip (Illumina, Inc., San Diego, CA, USA) (batch1) at the Wellcome Trust Sanger Institute, and with the Illumina Human Core Exome BeadChip (Illumina) (batch2) at the Wellcome Trust Sanger Institute and at the Broad Institute of MIT and Harvard (batch3). Genotype quality control thresholds have been previously described (He *et al*. 2016) and listed in Supporting Information Table S1. Pre‐phasing of the data was done with SHAPEIT2 (Delaneau *et al*. 2013). The pre‐phased genotype data were imputed with IMPUTE version 2.3.1 (Howie *et al*. 2009) using a combined reference panel consisting of 1000 Genomes Phase I (haplotype released in September 2013) and 1941 Finnish low‐pass whole genome sequences from the SISu project. The two panels consist of 37 878 799 and 13 625 209 variants, respectively. Following post‐imputation, exclusion criteria were applied for SNPs: effect allele frequency <0.01 and >0.99, SNP call rate < 0.95, HWE *P* < 1.0 × 10^−6^, and imputation info <0.8. For batches 1, 2 and 3, the number of variants that passed the quality control procedure was 521 529, 342 853 and 322 926, respectively. Quality controls and imputation for the GWAS data were done centrally at the Institute for Molecular Medicine Finland (FIMM), University of Helsinki, Helsinki, Finland.

### Replication analysis in the FinnTwin12 sample

We attempted to replicate the genome‐wide significant association signals detected with CPD on 16p and with MaxCigs24 on 3p and 16p, in a population‐based sample of young Finnish twins (FinnTwin12) born 1983–1987 (Kaprio 2006; Kaprio 2013). FinnTwin12 study has been approved by the IRB of Indiana University at Bloomington, Indiana, USA. The self‐reported CPD was assessed as a categorical variable of eight categories (1–2, 3–5, 6–10, 11–15, 16–19, 20–25, 26–39, ≥40 CPD during the period of heaviest smoking) at a mean age of 14.2 (standard deviation (SD) 0.1). In the analyses, we used class means of each category. MaxCigs24 was assessed and analyzed as a quantitative variable. Altogether 581 participants were ever smokers (smoked at least 100 cigarettes during lifetime) and were included in the analysis of CPD and MaxCigs24. DSM‐IV ND and NW phenotypes were not available in the FinnTwin12 sample.

Genotyping of the FinnTwin12 sample was done within the same genotyping batches as for the discovery sample, with identical quality controls and imputation procedures.

### Replication analysis in the sample drawn from the National FINRISK survey

We attempted to replicate the genome‐wide significant association signals detected with CPD on 16p in a sample that was drawn from a large population‐based study, the National FINRISK survey. The study has been initiated in 1972 and carried out since then every 5 years using independent, random and representative samples from four to six different parts of Finland depending on the year of the survey (Borodulin *et al*. 2015).

We used data from cohorts 1992, 1997, 2002, 2007 and 2012. The replication sample comprised of 6763 genotyped subjects (56 percent males) with self‐reported information on tobacco use and smoking amount. Mean age for the sample was 45 years. The smoking quantity trait was derived from quantitative self‐reported measure of cigarettes smoked per day (CPD), restricted to current smokers. The continuous CPD was further transformed to log scale (natural log) in order to roughly achieve a better correspondence of the distribution of the phenotype to the discovery sample's trait distribution. Comparison of the sample distributions between the discovery and replication samples is illustrated in Supporting Information Figure S1.

Genotyping of the replication sample was performed in several batches using Illumina 610‐quad BeadChip (Illumina, Inc., San Diego, CA, USA) and the Illumina Human Core Exome BeadChip (Illumina) at several genotyping centers which are listed in Supporting Information Table S2. Genotype quality control thresholds have also been listed in Table S2. Pre‐phasing of the data was done with Eagle version 2.3 (Loh *et al*. 2016). The pre‐phased genotype data were imputed with IMPUTE version 2.3.2 (Howie *et al*. 2009) using an all‐Finnish reference panel generated from the SISu project. Altogether, the reference panel consists of 15 490 261 variants from 2690 high‐pass whole genome sequences, and 184 117 variants from 5092 high‐pass‐whole exome sequences. The same post‐imputation exclusion criteria were applied for SNPs in the replication sample as in the discovery sample: effect allele frequency <0.01 and >0.99, SNP call rate < 0.95, HWE *P* < 1.0 × 10^−6^, and imputation info <0.8.

### Statistical analyses

#### GWAS analyses

The analyses were performed using the software tool genome‐wide efficient mixed‐model association (Zhou & Stephens, 2012). Allelic dosage data were used to account for genotype uncertainties. The genetic associations were modeled using a linear mixed model in which the phenotype was the dependent variable and the coded allele dose (represented by the posterior mean genotypes) was the independent variable. The model included age and sex as covariates. In addition, population stratification and relatedness within the sample were accounted for by the covariance matrix, which was determined by a relatedness matrix calculated from genome‐wide genotype data, representing genetic similarity across individuals. The number of markers included in the analyses was 9 469 131. *P*‐values below 5.0 × 10^−8^ were considered as genome‐wide significant, and *P*‐values below 5.0 × 10^−7^ were considered as approaching genome‐wide significance. For ease of interpretation of the results, beta coefficients are reported for the minor alleles.

Linear mixed models were used for both quantitative and binary traits, since the covariance structure of the data cannot be fully adjusted for in a logistic model. For binary traits, the obtained effect sizes were then transformed to the odds‐scale for more meaningful interpretations using a previously suggested formula (Pirinen *et al*. 2013). This has been shown to yield accurate estimates of the effect sizes when genetic effects are small, the case–control ratio is balanced and the minor allele frequency is above 0.05 (Pirinen *et al*. 2013). Odds ratios (OR) have been presented along with beta coefficients whenever at least one of the three criteria was met in order to facilitate interpretation of the effect sizes, but we emphasize that this must be done with caution.

#### Conditional analyses

Genomic loci exceeding genome‐wide significance were further targeted with conditional analyses to estimate the number of independent signals. We ran association analyses for loci of interest conditioning on the SNP with the lowest *P*‐value. The next signal was identified from the conditional analysis and included in the second round of conditional analyses. This process was repeated in an iterative fashion until no residual genome‐wide significant signal (*P* < 5.0 × 10^−8^) remained.

#### Replication analyses

For FinnTwin12‐replication sample, the analyses were performed using the same tool and method that was used for our discovery sample. For FINRISK‐replication sample, the analyses were performed using RVTEST (Zhan *et al*. 2016), which utilizes linear mixed model to generate associations between the phenotypic variable and genetic variable. The analyses were adjusted for age, sex and first 10 principal components.

### Annotation of genome‐wide significant SNPs

In order to infer the functional potential of the SNPs, we used publicly available databases to annotate the expression quantitative trait loci (eQTL) and methylation quantitative trait loci (meQTL) associated with the genome‐wide significant SNPs. Please refer to the Supporting Information Document S2 for detailed description of the methodology.

## Results

### Smoking quantity during the period of heaviest smoking

**Figure 1:**
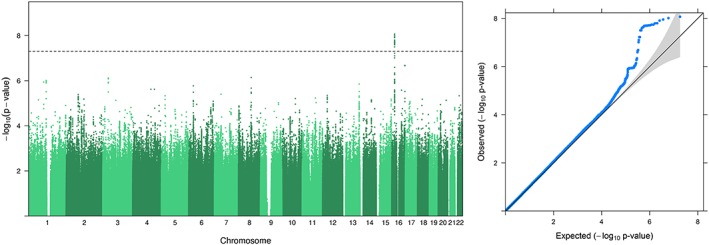
Manhattan and QQ plots of the GWAS results for CPD. Horizontal line in the Manhattan plot depicts the *P* < 5 × 10^−8^ threshold for genome‐wide significance. Genomic inflation factor λ = 1.026

**Figure 2:**
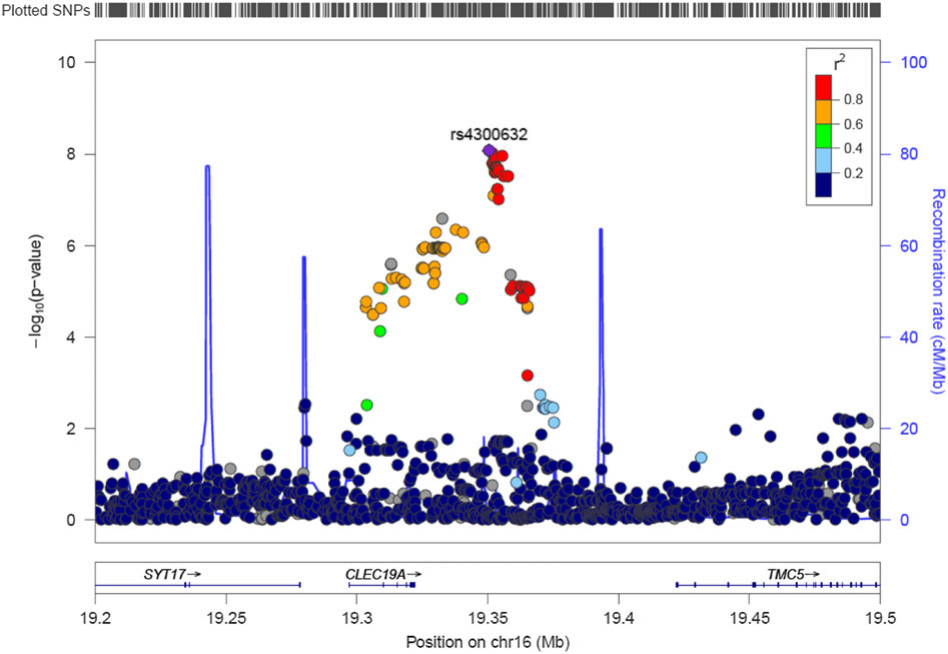
Regional plot of 16p12.3 results for CPD. The plot was generated with LocusZoom (Pruim *et al*. 2010), and the LD information has been obtained from hg19/1000 Genomes Nov 2014 EUR build

We detected genome‐wide significant association on 16p12.3 for CPD (min *P* = 8.5 × 10^−9^, beta = 4.75 for rs4300632) and MaxCigs24 (min *P* = 7.0 × 10^−9^, beta = 6.03 for rs2353663). Manhattan and QQ plots for CPD are presented in Figure 1. Results for MaxCigs24 were very similar to CPD and are available in Supporting Information Figure S2. For CPD, altogether 23 SNPs exceeded the genome‐wide significance threshold (Figure 2). The association signal emerged 28 kb from *CLEC19A* (C‐type lectin domain family 19 member A). For both CPD and MaxCigs24, conditional analysis revealed the presence of only one independent locus within the region.

Another genome‐wide significant locus was detected on 3p22.3 for MaxCigs24 (min *P* = 3.1 × 10^−8^, beta = 12.49 for rs73064179). The association signal peaks at an intergenic region, approximately 183 kb upstream of *STAC* (SH3 and cysteine rich domain). However, five additional SNPs in the region were approaching genome‐wide significance (rs56027566, rs12495177, rs73052216, rs73052223, rs73052229), located adjacent to *STAC*, the closest one being rs73052229 located approximately 10 kb upstream of the transcription start site (Figure S3). Conditional analysis suggested that the signal is driven by a single locus within the region. Top‐3 SNPs for each highlighted loci are presented in Table 2. Supplemental Tables S3 and S4 enclose the top‐100 SNP results for CPD and MaxCigs24, respectively.

**Table 2:** Top‐3 SNPs for each loci highlighted with associations reaching or approaching genome‐wide significance.

					CPD			MaxCigs24				MAF		Gene
Chr	BP	SNP^a^	A1	A2	*P* value^b^	*β* c	95% CI	*P* value b	*β* c		95% CI	Sample^e^	CEU f	
3p22.3	36238681	rs73064179	G	A	7.52E − 07	8.28	5.01–11.55	**3.07E − 08**	12.49		8.10–16.88	0.010	0.015	*STAC* (183 kb upstream)
	36347847	rs56027566	G	A	8.22E − 07	8.06	4.87–11.25	*1.01E* − *07*	11.73		7.44–16.02	0.011	0.025	*STAC* (74 kb upstream)
	36411991	rs73052229	C	T	3.14E − 06	7.57	4.40–10.75	*2.88E* − *07*	11.23		6.96–15.50	0.011	0.025	*STAC* (10 kb upstream)
16p12.3	19350508	rs4300632	T	A	**8.47E − 09**	4.75	3.14–6.36	**1.91E − 08**	6.17		4.03–8.31	0.046	0.040	*CLEC19A* (28 kb downstream)
	19351749	rs11074386	T	C	**9.83E − 09**	4.73	3.12–6.34	**2.08E − 08**	6.16		4.01–8.30	0.046	0.040	*CLEC19A* (29 kb downstream)
	19355577	rs11074388	A	G	**1.13E − 08**	4.39	2.90–5.88	**1.22E − 08**	5.83		3.83–7.83	0.055	0.040	*CLEC19A* (33 kb downstream)
					*NW symptom count*			*NW diagnosis*				*MAF*		*Gene*
Chr	BP	SNP^a^	A1	A2	*P* value^b^	*β* c	95% CI	*P* value^b^	*β* c	OR d	95% CI^d^	Sample^e^	CEU^f^	
2q21.3	135491290	rs74865979	G	T	3.66E − 06	0.54	0.31–0.77	*1.64E* − *07*	0.13	1.81	1.45–2.27	0.107	*NA*	*TMEM163* (intronic)
	135516997	rs62171406	G	A	*2.22E* − *07*	0.64	0.40–0.87	**2.10E − 09**	0.16	2.04	1.62–2.57	0.103	*NA*	*TMEM163* (intronic)
	135523638	rs75435861	T	A	1.32E − 06	0.61	0.36–0.86	*1.38E* − *07*	0.15	1.90	1.50–2.42	0.094	0.141	*TMEM163* (intronic)
11p15.5	983584	rs369708413	C	T	**6.58E − 08**	1.61	1.02–2.20	*3.02E* − *07*	0.34	4.36	2.49–7.6	0.017	0.005	*AP2A2* (intronic)
	993507	rs560149619	A	G	*1.68E* − *07*	1.50	0.95–2.05	1.05E − 06	0.31	3.79	2.22–6.45	0.017	0	*AP2A2* (intronic)
	1028665	rs201137338	G	C	*4.21E* − *07*	1.40	0.87–1.93	1.74E − 06	0.29	3.51	2.10–5.85	0.018	0	*MUC6* (missense: H524Q)
18q12.3	42839275	rs117354958	G	A	**3.55E − 08**	1.31	0.85–1.78	*4.57E* − *07*	0.27	3.12	2.01–4.85	0.024	0.066	*SLC14A2* (intronic)
	42849564	rs78012883	G	A	7.00E − 06	0.93	0.53–1.34	1.58E − 05	0.20	2.33	1.59–3.42	0.034	0.071	*SLC14A2* (intronic)
	42921854	rs112833408	T	C	4.75E − 05	1.03	0.54–1.53	2.42E − 05	0.24	2.75	1.72–4.40	0.024	0.045	*SLC14A2* (intronic)

Abbreviations: A1, effect allele (minor allele); A2, alternative allele (major allele); BP, base pair position according to Build37 of the human genome; CPD, cigarettes per day; Chr, chromosome; MaxCigs24, largest number of cigarettes ever‐smoked during a 24‐hour period; MAF, minor allele frequency; NW, nicotine withdrawal.

a rs‐number for the single‐nucleotide polymorphism (SNP).

b *p*‐value associated with beta coefficient.

c Beta coefficient.

d Cautious interpretation of the odds ratios and confidence intervals (CIs) is recommended as some of the assumptions (genetic effects are small, MAF < 0.05, case–control ratio is balanced) of the method used to calculate them are violated.

e Provided by the study sample genotypes (*n* = 2 063).

f Provided by Ensembl GRCh37 release 84—July 2016. Genome‐wide significant *p*‐values are underlined and highlighted in bold; *p*‐values approaching genome‐wide significance (*p* < 5.0 × 10^−7^) are highlighted in italics.

### DSM‐IV Nicotine Dependence

No genome‐wide significant association was detected for DSM‐IV ND. Manhattan and QQ plots for DSM‐IV ND diagnosis and DSM‐IV ND symptom count are presented in the Supplemental Figures S4 and S5, respectively. Top‐100 SNP results for DSM‐IV ND diagnosis and DSM‐IV ND symptom count are presented in Supplemental Tables S5 and S6, respectively.

### DSM‐IV Nicotine Withdrawal

DSM‐IV NW diagnosis showed genome‐wide significant association on 2q21.3 (min *P* = 2.1 × 10^−9^, beta = 0.16 for rs62171406), in an intron of *TMEM163* (transmembrane protein 163) (Supporting Information Figure S6). Manhattan and QQ plots are presented in Supplemental Figure S7. Conditional analysis suggested that the signal is driven by a single locus within the region.

**Figure 3:**
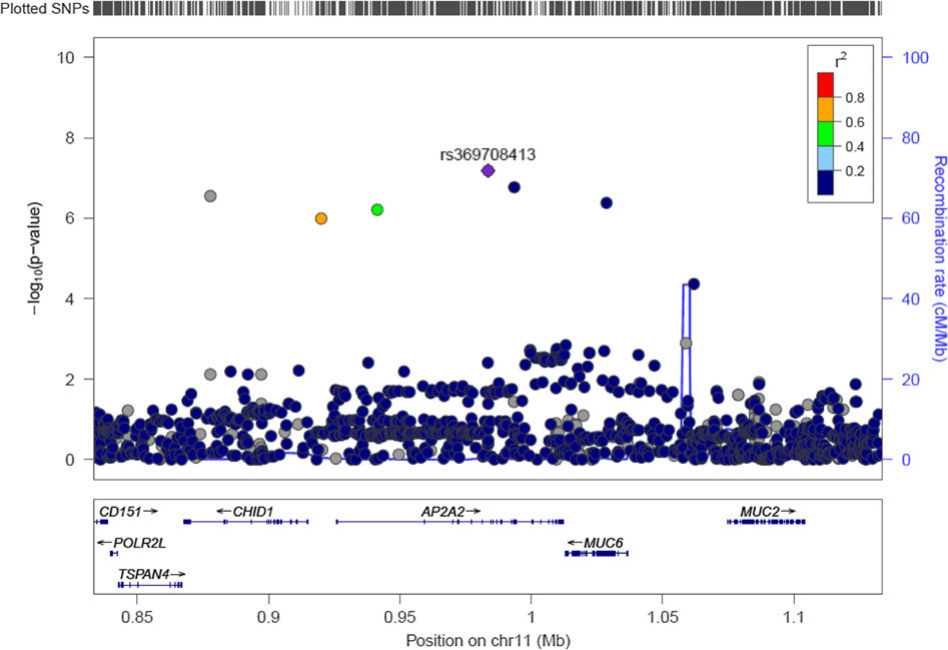
Regional plot of 11p15.5 results for continuous DSM‐IV nicotine withdrawal. The plot was generated with LocusZoom (Pruim *et al*. 2010), and the LD information has been obtained from hg19/1000 Genomes Nov 2014 EUR build

The results for DSM‐IV NW symptom count pinpointed loci on 11p15.5 and 18q12.3. Manhattan and QQ plots are presented in Supplemental Figure S8. On 11p15.5, an association signal approaching genome‐wide significance highlighted *AP2A2* (adaptor related protein complex 2 alpha 2 subunit) (min *P* = 6.6 × 10^−8^, beta = 1.61 for rs369708413) and *MUC6* (mucin 6, oligomeric mucus/gel‐forming) (min *P* = 4.2 × 10^−7^, beta = 1.40 for rs201137338) (Figure 3). *DRD4* (dopamine receptor D4) is located approximately 343 kb upstream from the 11p15.5 association locus but showed no significant association. On 18q12.3, a SNP in an intron of *SLC14A2* (solute carrier family 14 member 2) exceeded the genome‐wide significance threshold (*P* = 3.5 × 10^−8^, beta = 1.31 for rs117354958) (Figure S9). Top‐3 SNPs for each highlighted loci are presented in Table 2. Supplemental Tables S7 and S8 enclose the top‐100 SNP results for DSM‐IV NW diagnosis and DSM‐IV NW symptom count, respectively.

### Replication analyses for smoking quantity

The association between CPD and 16p12 locus was not replicated in the FinnTwin12 replication sample (*n* = 581). Also, no replication was observed with CPD on 16p12.3 with the FINRISK replication sample (*n* = 6763). The results for discovery and FINRISK‐replication samples concerning 16p12.3 region are described and compared in more detail in Supplemental Table S9.

### The CHRNA5‐CHRNA3‐CHRNB4 gene cluster

The most well‐established locus associated with smoking‐related traits, the *CHRNA5*‐*CHRNA3*‐*CHRNB4* gene cluster on 15q25.1, showed no genome‐wide significant association with any of the included traits. In our data, the locus was tagged by rs1051730—a SNP in perfect LD with the functional variant D398N (rs16969968). This SNP showed negligible association with CPD (*P* = 0.06, beta = 0.68), DSM‐IV ND symptom count (*P* = 0.001, beta = 0.20), and DSM‐IV NW symptom count (*P* = 0.009, beta = 0.19). DSM‐IV diagnosis of ND and NW showed similar results compared to the corresponding symptom counts.

We tested association between CPD and chromosome 15 in our FINRISK‐replication sample and detected significant association in and within close vicinity of the nicotinic receptor gene cluster (min *P* = 7.06 × 10^−11^, beta = 0.08 for rs8040868). Variant rs1051730, along with other highly correlated variants, provided significant association with CPD (*P* = 1.32 × 10^−9^, beta = 0.08). These results are illustrated in Supporting Information Figure S10. Chromosome 15 association results from discovery and FINRISK‐replication samples are further compared in Supporting Information Table S10.

### Annotation of genome‐wide significant SNPs

Since most of the 27 genome‐wide significant SNPs were in the intergenic or intronic regions, we annotated their functional potential using publicly available databases. According to Ensembl Variant Effect Predictor, *CLEC19A* SNP rs1004892 is present in an open chromatin region and may have regulatory potential (Supplemental Table S11). *CLEC19A* was insufficiently expressed in GTEx and BRAINEAC data and could not be tested for eQTLs. Instead, we tested *CLEC19A* SNPs as eQTLs for the flanking genes *SYT17* (18.5 kb upstream of *CLEC19A*) and *TMC5* (125 kb downstream of *CLEC19A*). No eQTLs were seen in GTEx blood‐derived data. In contrast, brain‐derived data from both GTEx and BRAINEAC showed several eQTLs: BRAINEAC data revealed 21 eQTLs for *SYT17* in frontal, temporal and occipital cortex, and two eQTLs for *TMC5* in cerebellar cortex, while GTEx data revealed 16 eQTLs for *SYT17* in cerebellar hemisphere and hypothalamus (all overlapping with the 21 eQTLs from BRAINEAC) (Supplemental Table S12). Further, GTEx data revealed eQTLs for rs62171406 in *TMEM163* in both blood and brain (frontal cortex).

We also examined the genome‐wide significant SNPs for meQTLs in blood‐ and brain‐derived data. No *cis*‐meQTLs were detected in the mQTLdb which contains blood derived data in mother–child pairs. However, the BIOSqtl browser reported rs62171406 as *cis*‐meQTLs with two *TMEM163* methylation probes in blood (Supplemental Table S13) (the same SNP was also identified as an eQTL in GTEx). None of the genome‐wide significant SNPs were reported as meQTLs in the fetal brain database.

## Discussion

Despite all the work invested in current genome‐wide approaches, the high heritability estimates of smoking behavior are nowhere near to be explained. This urges for more thorough scanning through the genome with alternative approaches. In this study, we performed a GWAS in a Finnish twin family sample (*n* = 1715) selected and enriched for smoking, with three distinct measures of nicotine addiction—smoking quantity, ND and NW. We aimed to improve imputation accuracy by combining the 1000 Genomes Phase I September 2013 release reference panel with a population‐specific reference panel of 1941 Finnish whole genome sequences, to allow for reliable scrutiny of low frequency variants.

Our study yielded genome‐wide significant association with CPD and MaxCigs24 on 16p12.3 near *CLEC19A*, and thus strengthened our previously reported GWAS findings (min *P* = 1.02 × 10^−7^) in a subset of 1114 individuals from the current sample (Loukola *et al*. 2014). *CLEC19A* has an unknown function and low expression levels in various tissues (gtexportal.org). It is plausible that the associating SNPs tag causal variants or regulatory motifs within this region. Interestingly, several of the genome‐wide significant SNPs were identified as eQTLs for neighboring genes *SYT17* and *TMC5* in publicly available brain tissue databases. However, no replication on 16p12.3 was observed in an independent adolescent Finnish twin sample (*n* = 581) FinnTwin12 replication sample, or in an independent Finnish population‐based sample (*n* = 6763) drawn from the National FINRISK Survey.

In the discovery sample, the detected effect sizes for locus 16p12.3 are impressive when compared to the modest effect sizes of the most well‐established smoking quantity locus on 15q25. However, due to the relatively low MAFs of the associating SNPs on 16p12.3, the population level impact is less notable than that of the robust smoking quantity locus on 15q25 (MAF about 0.34). Our discovery sample detected no genome‐wide significant association on 15q25, in line with our previous GWAS with a smaller but overlapping sample (Loukola *et al*. 2014). The effect size for D398N was consistent with prior reports of less than one CPD per allele. It is generally acknowledged that in order to detect association signal at the 15q25 locus (harboring the *CHRNA5‐CHRNA3‐CHRNB4* nAChR gene cluster), large samples are required, due to the small effect sizes of the associating variants. Our FINRISK‐replication sample provides ideal conditions for testing this dilemma, being a fairly large and independent sample (*n* = 6763). We detected significant association (*P* = 7.06 × 10^−11^, beta = 0.08 for rs8040868) on 15q25 in the FINRISK‐replication sample.

As the signal on 16p12.3 locus was not replicated, we cannot rule out the possibility of a false positive finding. However, 16p12.3 locus remains of interest due to evidence pointing to a co‐morbidity driven association. The locus has previously been linked to ADHD (Romanos *et al*. 2008). Also, nominally significant linkage with maximum number of cigarettes smoked (MaxCigs24) was found on 16p12.3 in a linkage meta‐analysis (Han *et al*. 2010). This locus was not found in a large‐scale GWAS meta‐analysis of CPD nor in a recent GWAS meta‐analysis of cotinine (Ware *et al*. 2016), the primary metabolite of nicotine which is a reliable biomarker of smoking quantity (Keskitalo *et al*. 2009). Thus, our signal on 16p12.3 may not be specific to smoking quantity but rather reflect co‐morbidity between ADHD liability or some other neuropsychiatric condition and smoking. Unfortunately, we were unable to test this hypothesis in the available cohorts.

In addition, it needs to be stressed that our discovery sample is highly enriched for heavy smoking, and thus, ND, and this phenotypic selection could have an entirely sample‐specific effect on the results based on genotypic drifting (1000 Genomes Project Consortium *et al*. 2015; Moltke *et al*. 2014). We observed a significant difference (*P* = 3.83 × 10^−5^) in the MAFs between the discovery and replication sample using a simple two‐sample Student's t‐test. The difference is described in Supporting Information Table S9.

Within the LD block on 16p12.3 harboring variants showing association with CPD stands an established transcription factor binding site (at chr16:19,328,414–19,328,427). This may provide one mechanism for the detected association. According to the UCSC Genome Browser (genome.ucsc.edu), this locus serves as a binding site for nuclear factor kappaB (NF‐κB) transcription factors. NF‐κB is a pleiotropic and highly conserved transcription factor family, which has roles in complex pathways regulating the developmental and synaptic plasticity, such as the neurotrophin signaling pathway (Mattson & Meffert 2006). Neurotrophins are a family of trophic factors involved in differentiation and survival of neural cells (Bibel & Barde 2000). Signals produced by this pathway have also been linked to mechanisms underlying learning, memory and drug addiction (Bolanos & Nestler 2004). The neurotrophin signaling pathway has previously been associated with smoking initiation, progression and cessation (Lang *et al*. 2007; The Tobacco and Genetics Consortium 2010; Wang & Li 2010), and the associations have mostly highlighted two members of the pathway: brain‐derived neurotrophic factor (*BDNF*) and neurotrophic tyrosine kinase receptor 2. In the current study, however, we detected no association with *BDNF* or neurotrophic tyrosine kinase receptor 2 with our studied phenotypes. The association of smoking with BDNF is primarily with initiation and the effect size modest.

The neutrotrophin signaling pathway was also highlighted in our GWAS of DSM‐IV NW symptom count. We detected an association signal approaching genome‐wide significance on 11p15.5 harboring *AP2A2* and *MUC6*. Interestingly, the 11p15 locus has previously shown genome‐wide significant linkage with DSM‐IV NW in a subset of 505 individuals from the current sample (Pergadia *et al*. 2009). The adaptor‐protein 2 plays a key role in clathrin‐mediated endocytosis (Smythe 2002), which is a major route for receptor distribution and internalization involved in the retrograde neurotrophin signaling pathway (Beattie *et al*. 2000). Evidence suggests that this process is involved in opiate drug, such as cocaine, addiction (Whistler *et al*. 1999). *MUC6* is upregulated by NFκB1 (Sakai *et al*. 2005), one of the NF‐κB proteins involved in the neurotrophin signaling pathway (Mattson & Meffert 2006). We detected no association with *DRD4* located on the 11p15.5 locus.

Besides findings related to the neurotrophin signaling pathway our analyses revealed other interesting signals. First, we detected genome‐wide significant association for MaxCigs24 on 3p22.3, in close vicinity of *STAC*, encoding a neuron‐specific protein consisting of a cysteine‐rich domain and a SH3 domain (Kawai *et al*. 1998). Little is known about the function of the gene. Elevated gene expression levels have been detected in artery tissues, mainly in the aorta, brain and lungs (gtexportal.org), all tissues affected by smoking and nicotine. Associating SNPs within the region are low‐frequency variants (MAF~ 0.01) with remarkably large effect sizes in this study (e.g. beta = 12.49 for rs73064179); however, the sample is moderately sized (*n* = 1715), which can lead to false positive findings. Second, a single SNP on 18q12.3 showed genome‐wide significant association with DSM‐IV NW symptom count. The signal emerges in an intron of *SLC14A2* which is a member of the urea transporter family. This finding, however, is not supported by other variants within the region. Third, we detected genome‐wide significant association for DSM‐IV NW diagnosis on 2q21.3, highlighting *TMEM163,* a zinc ion binder that shows high expression levels in the brain. The genome‐wide significant SNP in *TMEM163* (rs62171406) was identified as an eQTL in both blood and frontal cortex, and as a meQTL in whole blood, suggesting that this SNP can affect expression and methylation levels of *TMEM163*.

For the current study, we applied a stochastic approach by simultaneously utilizing the 1000Genomes Phase I reference panel and an all‐Finnish reference set based on 1941 Finnish whole genome sequences from the SISu project (sisuproject.fi). Owing to its population history of founding bottlenecks approximately 100 generations ago, the Finnish population offers substantial advantages in the study of rare and low frequency DNA variation by enabling more precise imputation of these variants. This approach showed improvement in our venture of finding susceptibility variants predisposing individuals to smoking behavior. As an example, our association of DSM‐IV NW symptom count on 11p15.5 is driven by a low‐frequency variant in *AP2A2* enriched in Finns. The MAF of the lead SNP (rs369708413) is 0.017 in the study sample, whereas in the general European population (Ensembl GRCh37 release 84—July 2016), it is 0.003, in other words, over five times smaller. This SNP is not included in the 1000 Genomes Phase I reference, and it was imputed to the data from the SISu reference panel. Had we only used the 1000 Genomes Phase I reference, many low‐frequency variants, including rs369708413, would have been left out from the analyses, as has been previously reported (Surakka *et al*. 2016). Comparison of results obtained using HapMap2 (rel#24 CEU—NCBI Build 36), 1000 Genomes Phase I (1000 Genomes Project Consortium *et al*. 2012) and 1000 Genomes Phase I + SISu (Surakka *et al*. 2016) imputed data in the discovery sample is presented in the Supporting Information Document S3.

To conclude, our study yielded genome‐wide significant association on 16p12.3 (near *CLEC19A*) for CPD. The associating SNPs were identified as eQTLs for neighboring genes *SYT17* and *TMC5*. However, more work is needed in order to verify the association, as the signal did not replicate. In addition, we detected an association signal approaching genome‐wide significance on 11p15.5 for DSM‐IV NW, in a locus previously linked to NW in a subset of individuals from the current sample (Pergadia *et al*. 2009). Our findings on both 16p12.3 and 11p15.5 highlight the neurotrophin signaling pathway. The role of neurotrophin signaling in nicotine addiction and co‐morbid traits remains to be confirmed and extended in further studies.

## Conflict of Interest

Prof. Kaprio and Dr Korhonen have provided consultation to Pfizer on nicotine dependence during 2011–2014 and 2011–2015, respectively.

## Authors Contribution

Hällfors J: Interpreted results for discovery sample and replication sample, wrote the manuscript with A. Loukola.

Palviainen T: Performed GWAS and secondary analyses.

Surakka I: Performed genotype data imputation.

Gupta R: Performed annotation analyses.

Buchwald J: Contributed to secondary statistical analyses.

Raevuori A: Co‐designed phenotype collection for the FinnTwin12 sample, consulting physician/ psychiatrist for FinnTwin12 sample.

Ripatti S: Supervised generation of NAG‐FIN genotype data.

Korhonen T: Further developed NAG‐FIN smoking‐related phenotypes.

Jousilahti P: Director of the National FINRISK Survey, provided expertise related to the sample.

Madden PAF: Co‐designed phenotype collection for the NAG‐FIN sample.

Kaprio J: NAG‐FIN sample PI, co‐designed phenotype and DNA collection for the NAG‐FIN sample, co‐PI for FinnTwin12 sample.

Loukola A: Designed the study, supervised analyses, interpreted results, wrote the manuscript with J. Hällfors.

## Supporting information


**Supplemental Table S1.** Discovery sample cohort supplementary information.
**Supplemental Table S2**. Replication sample cohort supplementary information.
**Supplemental Table S3**. Top‐100 SNP results for cigarettes per day (CPD).
**Supplemental Table S4**. Top‐100 SNP results for largest number of cigarettes ever‐smoked during a 24‐hour period (MaxCigs24).
**Supplemental Table S5**. Top‐100 SNP results for DSM‐IV nicotine dependence (ND) diagnosis.
**Supplemental Table S6**. Top‐100 SNP results for DSM‐IV nicotine dependence (ND) symptom count.
**Supplemental Table S7**. Top‐100 SNP results for DSM‐IV nicotine withdrawal (NW) diagnosis.
**Supplemental Table S8**. Top‐100 SNP results for DSM‐IV nicotine withdrawal (NW) symptom count.
**Supplemental Table S9**. Association results for 16p12.3 locus in the discovery and replication samples.
**Supplemental Table S10**. Association results for 15q25.1 locus harboring the cluster of nicotinic acetyl choline receptor genes *CHRNA5‐CHRNA3‐CHRNB4* in the discovery and replication samples.
**Supplemental Table S11**. Variant effect predictor results for the 27 genome‐wide significant SNPs identified across different phenotypes tested.
**Supplemental Table S12**. eQTLs identified among the 27 genome‐wide significant SNPs using brain‐derived data available at GTEx and BRAINEAC.
**Supplemental Table S13**. meQTLs observed among 27 genome‐wide significant SNPs using publicly available databases.
**Supplemental Figure S1.** CPD distributions for discovery sample (*n* = 1715) (A) and replication sample (*n* = 6763) (B).
**Supplemental Figure S2**. Manhattan and QQ plots of the GWAS results for MaxCigs24. Horizontal line in the Manhattan plot depicts the *P* < 5 × 10^−8^ threshold for genome‐wide significance. Genomic inflation factor λ = 1.008.
**Supplemental Figure S3**. Regional plot of 3p22.3 results for MaxCigs24. The plot was generated with LocusZoom (Pruim *et al*. 2010), and the LD information has been obtained from hg19/1000 Genomes Nov 2014 EUR build.
**Supplemental Figure S4**. Manhattan and QQ plots of the GWAS results for DSM‐IV ND diagnosis. Horizontal line in the Manhattan plot depicts the *P* < 5 × 10^−8^ threshold for genome‐wide significance. Genomic inflation factor λ = 1.017.
**Supplemental Figure S5**. Manhattan and QQ plots of the GWAS results for DSM‐IV ND symptom count. Horizontal line in the Manhattan plot depicts the *P* < 5 × 10^−8^ threshold for genome‐wide significance. Genomic inflation factor λ = 1.01.
**Supplemental Figure S6**. Regional plot of 2q21.3 results for DSM‐IV NW diagnosis. The plot was generated with LocusZoom (Pruim *et al*. 2010), and the LD information has been obtained from hg19/1000 Genomes Nov 2014 EUR build.
**Supplemental Figure S7**. Manhattan and QQ plots of the GWAS results for DSM‐IV NW diagnosis. Horizontal line in the Manhattan plot depicts the *P* < 5 × 10^−8^ threshold for genome‐wide significance. Genomic inflation factor λ = 1.012.
**Supplemental Figure S8**. Manhattan and QQ plots of the GWAS results for DSM‐IV NW symptom count. Horizontal line in the Manhattan plot depicts the *P* < 5 × 10^−8^ threshold for genome‐wide significance. Genomic inflation factor λ = 1.017.
**Supplemental Figure S9**. Regional plot of 18q12.3 results for DSM‐IV NW symptom count. The plot was generated with LocusZoom (Pruim *et al*. 2010), and the LD information has been obtained from hg19/1000 Genomes Nov 2014 EUR build.
**Supplemental Figure S10**. Regional plot of 15q25.1 results for CPD in the replication sample (*n* = 6763). The plot was generated with LocusZoom (Pruim *et al*. 2010), and the LD information has been obtained from hg19/1000 Genomes Nov 2014 EUR build.
**Supplemental Document S1**. DSM‐IV criteria for nicotine dependence (ND) and nicotine withdrawal (NW), as described by APA in 1994.
**Supplemental Document S2**. Annotation of genome‐wide significant SNPs, a section included in the Materials and methods.
**Supplemental Document S3**. Includes Supplemental Figure S10. Comparing the association results of 16p12.3 and CPD in three data sets (from the same sample) with different imputation reference panels: (A) HapMap2, (B) 1000Genomes phase 1 and (C) 1000Genomes phase 1 + SISu. Number of SNPs within the region included in the analysis: (A) 526, (B) 1282 and (C) 1304. Sample size in each data set: (A) *n* = 1105, (B) *n* = 1715 and (C) *n* = 1715.
