# Supplementary material for: Genome‐wide association study in Finnish twins highlights the connection between nicotine addiction and neurotrophin signaling pathway
Source: Addict Biol. 2018 Mar 13;24(3):549–61. doi: 10.1111/adb.12618 (PMC6519128; doi:10.1111/adb.12618)
Supplement: Supplementary file 1 — Supplemental Table S1. Discovery sample cohort supplementary information. Supplemental Table S2. Replication sample cohort supplementary information. Supplemental Table S3. Top‐100 SNP results for cigarettes per day (CPD). Supplemental Table S4. Top‐100 SNP results for largest number of cigarettes ever‐smoked during a 24‐hour period (MaxCigs24). Supplemental Table S5. Top‐100 SNP results for DSM‐IV nicotine dependence (ND) diagnosis. Supplemental Table S6. Top‐100 SNP results for DSM‐IV nicotine dependence (ND) symptom count. Supplemental Table S7. Top‐100 SNP results for DSM‐IV nicotine withdrawal (NW) diagnosis. Supplemental Table S8. Top‐100 SNP results for DSM‐IV nicotine withdrawal (NW) symptom count. Supplemental Table S9. Association results for 16p12.3 locus in the discovery and replication samples. Supplemental Table S10. Association results for 15q25.1 locus harboring the cluster of nicotinic acetyl choline receptor genes CHRNA5‐CHRNA3‐CHRNB4 in the discovery and replication samples. Supplemental Table S11. Variant effect predictor results for the 27 genome‐wide significant SNPs identified across different phenotypes tested. Supplemental Table S12. eQTLs identified among the 27 genome‐wide significant SNPs using brain‐derived data available at GTEx and BRAINEAC. Supplemental Table S13. meQTLs observed among 27 genome‐wide significant SNPs using publicly available databases. Supplemental Figure S1. CPD distributions for discovery sample (n = 1715) (A) and replication sample (n = 6763) (B). Supplemental Figure S2. Manhattan and QQ plots of the GWAS results for MaxCigs24. Horizontal line in the Manhattan plot depicts the P < 5 × 10^−8^ threshold for genome‐wide significance. Genomic inflation factor λ = 1.008. Supplemental Figure S3. Regional plot of 3p22.3 results for MaxCigs24. The plot was generated with LocusZoom (Pruim et al. 2010), and the LD information has been obtained from hg19/1000 Genomes Nov 2014 EUR build. Supplemental Figure S4. Manhattan and QQ [file ADB-24-549-s001.zip › ADB_12618_supp-0001-Document S1.pdf]

Supplemental Document 1.

DSM-IV Criteria for nicotine dependence (ND) and nicotine withdrawal (NW) – as described by the American Psychiatric Association in 1994 (American Psychiatric Association. (1994). Diagnostic and statistical manual of mental disorders: DSM-IV (4th ed.). Washington, DC: American Psychiatric Association.)

DSM-IV ND symptoms:

(A maladaptive pattern of nicotine use, leading to clinically significant impairment or distress, *as manifested by three (or more) of the following* seven symptoms occurring at the same time within a 12-month period)

1. Tolerance, as defined by either of the following:

   a. absence of nausea, dizziness, and other characteristic symptoms despite using substantial amounts of nicotine.
   b. diminished effect observed with continued use of the same amount of nicotine-containing products.

2. Withdrawal as manifested by either of the following:

   a. the characteristic withdrawal syndrome for nicotine (refer to Criteria A and B of the criteria sets for Withdrawal from the specific substances)
   b. nicotine (or a closely related) substance is taken to relieve or avoid withdrawal symptoms.

3. Nicotine is often taken in larger amounts or over a longer period than was intended.

4. There is a persistent desire or unsuccessful efforts to cut down or control nicotine use.

5. A great deal of time is spent in activities necessary to obtain nicotine (e.g., visiting multiple doctors or driving long distances), use nicotine (e.g., chain-smoking), or recover from its effects.

6. Important social, occupational, or recreational activities are given up or reduced because of nicotine.

7. Nicotine use is continued despite knowledge of having a persistent or recurrent physical or psychological problem that is likely to have been caused or exacerbated by nicotine.

DSM-IV NW symptoms:

The NW diagnosis requires at least four out of eight symptoms within 24 hours after an abrupt cessation of nicotine use or deduction in the amount of nicotine use.

Diagnostic criteria for nicotine withdrawal:

A) Daily use of nicotine for at least several weeks, and,

B) Abrupt cessation of nicotine use, or reduction in the amount of nicotine used, followed within 24 hours by four or more of the following signs:

a. Irritability, frustration, or anger

b. Anxiety,

c. Difficulty concentrating,

d. Restlessness

e. Decreased heart rate

f. Increased appetite or weight gain

g. Dysphoric or depressed mood

h. Insomnia
