# Supplementary material for: Genome‐wide association study in Finnish twins highlights the connection between nicotine addiction and neurotrophin signaling pathway
Source: Addict Biol. 2018 Mar 13;24(3):549–61. doi: 10.1111/adb.12618 (PMC6519128; doi:10.1111/adb.12618)
Supplement: Supplementary file 1 — Supplemental Table S1. Discovery sample cohort supplementary information. Supplemental Table S2. Replication sample cohort supplementary information. Supplemental Table S3. Top‐100 SNP results for cigarettes per day (CPD). Supplemental Table S4. Top‐100 SNP results for largest number of cigarettes ever‐smoked during a 24‐hour period (MaxCigs24). Supplemental Table S5. Top‐100 SNP results for DSM‐IV nicotine dependence (ND) diagnosis. Supplemental Table S6. Top‐100 SNP results for DSM‐IV nicotine dependence (ND) symptom count. Supplemental Table S7. Top‐100 SNP results for DSM‐IV nicotine withdrawal (NW) diagnosis. Supplemental Table S8. Top‐100 SNP results for DSM‐IV nicotine withdrawal (NW) symptom count. Supplemental Table S9. Association results for 16p12.3 locus in the discovery and replication samples. Supplemental Table S10. Association results for 15q25.1 locus harboring the cluster of nicotinic acetyl choline receptor genes CHRNA5‐CHRNA3‐CHRNB4 in the discovery and replication samples. Supplemental Table S11. Variant effect predictor results for the 27 genome‐wide significant SNPs identified across different phenotypes tested. Supplemental Table S12. eQTLs identified among the 27 genome‐wide significant SNPs using brain‐derived data available at GTEx and BRAINEAC. Supplemental Table S13. meQTLs observed among 27 genome‐wide significant SNPs using publicly available databases. Supplemental Figure S1. CPD distributions for discovery sample (n = 1715) (A) and replication sample (n = 6763) (B). Supplemental Figure S2. Manhattan and QQ plots of the GWAS results for MaxCigs24. Horizontal line in the Manhattan plot depicts the P < 5 × 10^−8^ threshold for genome‐wide significance. Genomic inflation factor λ = 1.008. Supplemental Figure S3. Regional plot of 3p22.3 results for MaxCigs24. The plot was generated with LocusZoom (Pruim et al. 2010), and the LD information has been obtained from hg19/1000 Genomes Nov 2014 EUR build. Supplemental Figure S4. Manhattan and QQ [file ADB-24-549-s001.zip › ADB_12618_supp-0002-Document S2.pdf]

Suppelemental Document 2: Annotation of genome-wide significant SNPs (Materials and methods)

In order to infer the functional potential of the SNPs we used the Ensembl Variant Effect Predictor (McLaren et al. 2016). To test whether the SNPs affect expression of nearby genes we performed expression quantitative trait loci (eQTL) analyses utilizing blood- and brain- tissue derived data available at the Genotype-Tissue Expression (GTEx) database (GTEx Consortium 2015) and brain-tissue derived data at the Brain eQTL Almanac (BRAINEAC) database (Ramasamy et al. 2014). To test whether the SNPs affect methylation levels of nearby genes we utilized blood-derived methylation quantitative trait loci (meQTL) data at the mQTLdb (mqtldb.org) (Gaunt et al. 2016) and the BIOSqtl browser (genenetwork.nl/biosqtlbrowser) (Bonder et al. 2016) (only epigenome-wide significant meQTLs listed) as well as meQTL data derived from fetal brain (epigenetics.essex.ac.uk/mQTL) (Hannon et al. 2016) (only epigenome-wide significant meQTLs listed).

References:

Bonder MJ, Luijk R, Zhernakova DV, Moed M, Deelen P, Vermaat M, van Iterson M, van Dijk F, van Galen M, Bot J, Slieker RC, Jhamai PM, Verbiest M, Suchiman HE, Verkerk M, van der Breggen R, van Rooij J, Lakenberg N, Arindrarto W, Kielbasa SM, Jonkers I, van 't Hof P, Nooren I, Beekman M, Deelen J, van Heemst D, Zhernakova A, Tigchelaar EF, Swertz MA, Hofman A, Uitterlinden AG, Pool R, van Dongen J, Hottenga JJ, Stehouwer CD, van der Kallen CJ, Schalkwijk CG, van den Berg LH, van Zwet EW, Mei H, Li Y, Lemire M, Hudson TJ, BIOS Consortium, Slagboom PE, Wijmenga C, Veldink JH, van Greevenbroek MM, van Duijn CM, Boomsma DI, Isaacs A, Jansen R, van Meurs JB, 't Hoen PA, Franke L, Heijmans BT (2016) Disease variants alter transcription factor levels and methylation of their binding sites. *Nat Genet* **49**: 131–138.

Gaunt TR, Shihab HA, Hemani G, Min JL, Woodward G, Lyttleton O, Zheng J, Duggirala A, McArdle WL, Ho K, Ring SM, Evans DM, Davey Smith G, Relton CL (2016) Systematic identification of genetic influences on methylation across the human life course. *Genome Biol* **17**:61-016-0926-z.

GTEx Consortium (2015) Human genomics. The Genotype-Tissue Expression (GTEx) pilot analysis: multitissue gene regulation in humans. *Science* **348**:648-660.

Hannon E, Spiers H, Viana J, Pidsley R, Burrage J, Murphy TM, Troakes C, Turecki G, O'Donovan MC, Schalkwyk LC, Bray NJ, Mill J (2016) Methylation QTLs in the developing brain and their enrichment in schizophrenia risk loci. *Nat Neurosci* **19**:48-54.

McLaren W, Gil L, Hunt SE, Riat HS, Ritchie GR, Thormann A, Flicek P, Cunningham F (2016) The Ensembl Variant Effect Predictor. *Genome Biol* **17**:122-016-0974-4.

Ramasamy A, Trabzuni D, Guelfi S, Varghese V, Smith C, Walker R, De T, UK Brain Expression Consortium, North American Brain Expression Consortium, Coin L, de Silva R, Cookson MR, Singleton AB, Hardy J, Ryten M, Weale ME (2014) Genetic variability in the regulation of gene expression in ten regions of the human brain. *Nat Neurosci* **17**:1418-1428.
