# Supplementary material for: Genome‐wide association study in Finnish twins highlights the connection between nicotine addiction and neurotrophin signaling pathway
Source: Addict Biol. 2018 Mar 13;24(3):549–61. doi: 10.1111/adb.12618 (PMC6519128; doi:10.1111/adb.12618)
Supplement: Supplementary file 1 — Supplemental Table S1. Discovery sample cohort supplementary information. Supplemental Table S2. Replication sample cohort supplementary information. Supplemental Table S3. Top‐100 SNP results for cigarettes per day (CPD). Supplemental Table S4. Top‐100 SNP results for largest number of cigarettes ever‐smoked during a 24‐hour period (MaxCigs24). Supplemental Table S5. Top‐100 SNP results for DSM‐IV nicotine dependence (ND) diagnosis. Supplemental Table S6. Top‐100 SNP results for DSM‐IV nicotine dependence (ND) symptom count. Supplemental Table S7. Top‐100 SNP results for DSM‐IV nicotine withdrawal (NW) diagnosis. Supplemental Table S8. Top‐100 SNP results for DSM‐IV nicotine withdrawal (NW) symptom count. Supplemental Table S9. Association results for 16p12.3 locus in the discovery and replication samples. Supplemental Table S10. Association results for 15q25.1 locus harboring the cluster of nicotinic acetyl choline receptor genes CHRNA5‐CHRNA3‐CHRNB4 in the discovery and replication samples. Supplemental Table S11. Variant effect predictor results for the 27 genome‐wide significant SNPs identified across different phenotypes tested. Supplemental Table S12. eQTLs identified among the 27 genome‐wide significant SNPs using brain‐derived data available at GTEx and BRAINEAC. Supplemental Table S13. meQTLs observed among 27 genome‐wide significant SNPs using publicly available databases. Supplemental Figure S1. CPD distributions for discovery sample (n = 1715) (A) and replication sample (n = 6763) (B). Supplemental Figure S2. Manhattan and QQ plots of the GWAS results for MaxCigs24. Horizontal line in the Manhattan plot depicts the P < 5 × 10^−8^ threshold for genome‐wide significance. Genomic inflation factor λ = 1.008. Supplemental Figure S3. Regional plot of 3p22.3 results for MaxCigs24. The plot was generated with LocusZoom (Pruim et al. 2010), and the LD information has been obtained from hg19/1000 Genomes Nov 2014 EUR build. Supplemental Figure S4. Manhattan and QQ [file ADB-24-549-s001.zip › ADB_12618_supp-0009-FIGURE S6.pdf]

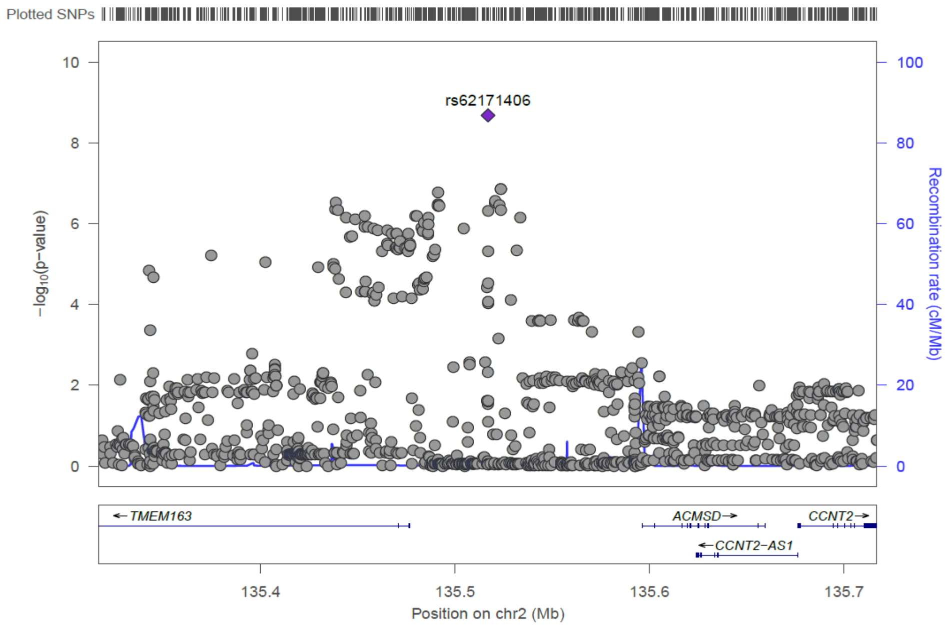

FIGURE S6. Regional plot of 2q21.3 results for DSM-IV NW diagnosis. The plot was generated with LocusZoom (Pruim *et al*. 2010), and the LD information has been obtained from hg19/1000 Genomes Nov 2014 EUR build.
