# Supplementary material for: Genome‐wide association study in Finnish twins highlights the connection between nicotine addiction and neurotrophin signaling pathway
Source: Addict Biol. 2018 Mar 13;24(3):549–61. doi: 10.1111/adb.12618 (PMC6519128; doi:10.1111/adb.12618)
Supplement: Supplementary file 1 — Supplemental Table S1. Discovery sample cohort supplementary information. Supplemental Table S2. Replication sample cohort supplementary information. Supplemental Table S3. Top‐100 SNP results for cigarettes per day (CPD). Supplemental Table S4. Top‐100 SNP results for largest number of cigarettes ever‐smoked during a 24‐hour period (MaxCigs24). Supplemental Table S5. Top‐100 SNP results for DSM‐IV nicotine dependence (ND) diagnosis. Supplemental Table S6. Top‐100 SNP results for DSM‐IV nicotine dependence (ND) symptom count. Supplemental Table S7. Top‐100 SNP results for DSM‐IV nicotine withdrawal (NW) diagnosis. Supplemental Table S8. Top‐100 SNP results for DSM‐IV nicotine withdrawal (NW) symptom count. Supplemental Table S9. Association results for 16p12.3 locus in the discovery and replication samples. Supplemental Table S10. Association results for 15q25.1 locus harboring the cluster of nicotinic acetyl choline receptor genes CHRNA5‐CHRNA3‐CHRNB4 in the discovery and replication samples. Supplemental Table S11. Variant effect predictor results for the 27 genome‐wide significant SNPs identified across different phenotypes tested. Supplemental Table S12. eQTLs identified among the 27 genome‐wide significant SNPs using brain‐derived data available at GTEx and BRAINEAC. Supplemental Table S13. meQTLs observed among 27 genome‐wide significant SNPs using publicly available databases. Supplemental Figure S1. CPD distributions for discovery sample (n = 1715) (A) and replication sample (n = 6763) (B). Supplemental Figure S2. Manhattan and QQ plots of the GWAS results for MaxCigs24. Horizontal line in the Manhattan plot depicts the P < 5 × 10^−8^ threshold for genome‐wide significance. Genomic inflation factor λ = 1.008. Supplemental Figure S3. Regional plot of 3p22.3 results for MaxCigs24. The plot was generated with LocusZoom (Pruim et al. 2010), and the LD information has been obtained from hg19/1000 Genomes Nov 2014 EUR build. Supplemental Figure S4. Manhattan and QQ [file ADB-24-549-s001.zip › ADB_12618_supp-0022-Table S9.pdf]

Supplemental Table S9. Association results for 16p12.3 locus in the discovery and replication samples.

Chr	BP[c]	SNP[d]	A1[e]	A2[f]	**Discovery sample (n=1715) [a]**				**Replication sample (n=6763) [b]**			
					p value[g]	*β*[h]	SE	MAF[i]	*p* value[g]	*β*[h]	SE	MAF[i]
16	19350508	rs4300632	T	A	**8.47E-09**	4.75	0.82	0.046	0.30	-0.03	0.032	0.042
16	19351749	rs11074386	T	C	**9.83E-09**	4.73	0.82	0.046	0.30	-0.03	0.032	0.042
16	19351837	rs11074387	G	A	**1.57E-08**	4.36	0.77	0.055	0.28	-0.03	0.028	0.051
16	19352221	rs4782229	T	C	**1.62E-08**	4.35	0.77	0.055	*NA*	*NA*	*NA*	*NA*
16	19352293	rs755385	T	C	8.01E-08	4.06	0.75	0.057	0.33	-0.03	0.027	0.056
16	19352463	rs916077	A	G	**1.80E-08**	4.34	0.77	0.055	0.28	-0.03	0.028	0.051
16	19352499	rs2353661	A	C	**1.81E-08**	4.34	0.77	0.055	0.28	-0.03	0.028	0.051
16	19352684	rs2353662	C	T	**2.57E-08**	4.37	0.78	0.055	0.26	-0.03	0.028	0.051
16	19352685	rs2353663	T	G	**2.44E-08**	4.37	0.78	0.055	0.28	-0.03	0.028	0.051
16	19352688	rs2353664	G	A	**2.26E-08**	4.39	0.78	0.055	0.28	-0.03	0.028	0.051
16	19353064	rs916078	T	C	**1.22E-08**	4.70	0.82	0.046	0.32	-0.03	0.032	0.042
16	19353107	rs139902244	ATTATTACCCC	A	**1.90E-08**	4.33	0.77	0.055	0.28	-0.03	0.028	0.051
16	19353159	rs762761	A	G	**1.93E-08**	4.33	0.77	0.055	0.28	-0.03	0.028	0.051
16	19353246	rs762762	C	T	**1.96E-08**	4.33	0.77	0.055	0.28	-0.03	0.028	0.051
16	19353328	rs4238673	C	A	**1.99E-08**	4.33	0.77	0.055	0.29	-0.03	0.028	0.051
16	19353409	rs4782232	G	A	**2.00E-08**	4.33	0.77	0.055	0.28	-0.03	0.028	0.051
16	19353429	rs4782233	C	T	**2.03E-08**	4.30	0.76	0.056	0.28	-0.03	0.028	0.051
16	19353430	rs4782234	T	C	**2.03E-08**	4.30	0.76	0.056	0.28	-0.03	0.028	0.051
16	19353688	rs2213278	A	G	5.96E-08	4.28	0.79	0.053	0.28	-0.03	0.028	0.051
16	19353731	rs2213279	A	G	5.99E-08	4.28	0.79	0.053	0.28	-0.03	0.028	0.051
16	19353940	rs2213280	A	G	**2.09E-08**	4.32	0.77	0.055	0.28	-0.03	0.028	0.051
16	19354089	rs2227005	T	C	**2.32E-08**	4.31	0.77	0.055	0.29	-0.03	0.028	0.051
16	19354175	rs2353668	T	G	9.75E-08	4.04	0.75	0.058	0.29	-0.03	0.028	0.051
16	19355577	rs11074388	A	G	**1.13E-08**	4.39	0.76	0.055	0.29	-0.03	0.028	0.051
16	19356105	rs1004892	G	C	**3.14E-08**	4.27	0.77	0.054	0.29	-0.03	0.028	0.051
16	19357701	rs11406076	G	GA	**3.14E-08**	4.27	0.77	0.054	0.29	-0.03	0.028	0.051
16	19357771	rs1004928	G	A	**3.14E-08**	4.27	0.77	0.054	0.28	-0.03	0.028	0.051

Note: A = allele; BP = base pair; Chr = chromosome; MAF = minor allele frequency (calculated from the data); SE = standard error;

[a] In the statistical analyses of the CPD variable, original categorical observations were replaced with class means of CPD (1.5, 3.5, 8, 13, 17.5, 22.5, 32.5, and 45 cigarettes per day, respectively). Regression coefficients can therefore be interpreted as the average change in number of cigarettes smoked per day when the number of minor allele is increased by one.

[b] Continuous CPD coded as a log-transformed (natural log) value

[c] base pair position according to Build37 of the human genome

[d] rs-number for the single-nucleotide polymorphism (SNP)

[e] effect allele (minor allele)

[f] alternative allele (major allele)

[g] p-value associated with beta

[h] beta coded against the reference allele

[i] Diffrences in the MAFs of these 26 variants described in the table between the discovery and replication samples were tested using a simple Two Sample student's t-test. According to the test, MAFs differ significantly from each other (*P* = 3.834e-05) with mean allele frequency difference 0.003846.
