# Supplementary material for: Genome‐wide association study in Finnish twins highlights the connection between nicotine addiction and neurotrophin signaling pathway
Source: Addict Biol. 2018 Mar 13;24(3):549–61. doi: 10.1111/adb.12618 (PMC6519128; doi:10.1111/adb.12618)
Supplement: Supplementary file 1 — Supplemental Table S1. Discovery sample cohort supplementary information. Supplemental Table S2. Replication sample cohort supplementary information. Supplemental Table S3. Top‐100 SNP results for cigarettes per day (CPD). Supplemental Table S4. Top‐100 SNP results for largest number of cigarettes ever‐smoked during a 24‐hour period (MaxCigs24). Supplemental Table S5. Top‐100 SNP results for DSM‐IV nicotine dependence (ND) diagnosis. Supplemental Table S6. Top‐100 SNP results for DSM‐IV nicotine dependence (ND) symptom count. Supplemental Table S7. Top‐100 SNP results for DSM‐IV nicotine withdrawal (NW) diagnosis. Supplemental Table S8. Top‐100 SNP results for DSM‐IV nicotine withdrawal (NW) symptom count. Supplemental Table S9. Association results for 16p12.3 locus in the discovery and replication samples. Supplemental Table S10. Association results for 15q25.1 locus harboring the cluster of nicotinic acetyl choline receptor genes CHRNA5‐CHRNA3‐CHRNB4 in the discovery and replication samples. Supplemental Table S11. Variant effect predictor results for the 27 genome‐wide significant SNPs identified across different phenotypes tested. Supplemental Table S12. eQTLs identified among the 27 genome‐wide significant SNPs using brain‐derived data available at GTEx and BRAINEAC. Supplemental Table S13. meQTLs observed among 27 genome‐wide significant SNPs using publicly available databases. Supplemental Figure S1. CPD distributions for discovery sample (n = 1715) (A) and replication sample (n = 6763) (B). Supplemental Figure S2. Manhattan and QQ plots of the GWAS results for MaxCigs24. Horizontal line in the Manhattan plot depicts the P < 5 × 10^−8^ threshold for genome‐wide significance. Genomic inflation factor λ = 1.008. Supplemental Figure S3. Regional plot of 3p22.3 results for MaxCigs24. The plot was generated with LocusZoom (Pruim et al. 2010), and the LD information has been obtained from hg19/1000 Genomes Nov 2014 EUR build. Supplemental Figure S4. Manhattan and QQ [file ADB-24-549-s001.zip › ADB_12618_supp-0023-Table S10.pdf]

Supplemental Table S10. Association results for 15q25.1 locus harboring the cluster of nicotinic acetyl choline receptor genes *CHRNA5-CHRNA3-CHRNB4* in the discovery and replication samples.

					Discovery sample (n=1715) [a]				Replication sample (n=6763) [b]			
Chr	BP[c]	SNP[d]	A1[e]	A2[f]	*p* value[g]	β[h]	SE	MAF	*p* value[g]	β[h]	SE	MAF
15	78766250	rs1504550	G	A	0.15	0.532	0.37	0.35	**1.49E-09**	0.080	0.01	0.31
15	78772676	rs17484524	G	A	0.15	0.532	0.37	0.35	**1.62E-09**	0.080	0.01	0.31
15	78783277	rs11858836	A	G	0.15	0.528	0.37	0.35	**1.65E-09**	0.080	0.01	0.31
15	78849779	rs72740955	T	C	0.09	0.620	0.36	0.37	**6.27E-10**	0.081	0.01	0.33
15	78851615	rs2036527	A	G	0.07	0.656	0.36	0.37	**6.49E-10**	0.081	0.01	0.33
15	78857939	rs55853698	G	T	0.08	0.645	0.36	0.37	**5.32E-10**	0.081	0.01	0.33
15	78857986	rs55781567	G	C	0.07	0.664	0.36	0.37	**1.14E-09**	0.080	0.01	0.33
15	78862064	rs11633958	T	C	0.09	0.606	0.36	0.37	**8.57E-10**	0.080	0.01	0.33
15	78862453	rs7172118	A	C	0.10	0.597	0.36	0.37	**8.91E-10**	0.080	0.01	0.33
15	78865197	rs17486195	G	A	0.10	0.601	0.36	0.37	**8.70E-10**	0.080	0.01	0.33
15	78866445	rs140330585	A	G	0.12	0.559	0.36	0.37	**1.27E-09**	0.080	0.01	0.33
15	78867482	rs17486278	C	A	0.10	0.594	0.36	0.37	**8.71E-10**	0.080	0.01	0.33
15	78868636	rs72740964	A	G	0.09	0.611	0.36	0.37	**8.72E-10**	0.080	0.01	0.33
15	78873993	rs7180002	T	A	0.09	0.616	0.36	0.37	**8.75E-10**	0.080	0.01	0.33
15	78877381	rs56390833	A	C	0.09	0.613	0.36	0.37	**4.05E-10**	0.082	0.01	0.33
15	78878541	rs951266	A	G	0.09	0.613	0.36	0.37	**7.71E-10**	0.080	0.01	0.33
15	78886198	rs8192482	T	C	0.09	0.620	0.36	0.37	**1.24E-09**	0.080	0.01	0.32
15	78886947	rs4887067	A	G	0.09	0.619	0.36	0.37	**1.25E-09**	0.080	0.01	0.32
15	78894339	rs1051730	A	G	0.06	0.680	0.36	0.37	**1.32E-09**	0.080	0.01	0.32
15	78896129	rs1317286	G	A	0.11	0.575	0.36	0.37	**1.32E-09**	0.080	0.01	0.32
15	78898932	rs55676755	G	C	0.07	0.654	0.36	0.37	**1.39E-09**	0.079	0.01	0.32
15	78899003	rs56077333	A	C	0.04	0.745	0.37	0.36	**1.31E-09**	0.080	0.01	0.32
15	78900701	rs138544659	G	T	0.08	0.645	0.37	0.36	**1.34E-09**	0.079	0.01	0.32
15	78900908	rs147144681	T	C	0.05	0.706	0.37	0.36	**1.38E-09**	0.079	0.01	0.32
15	78901173	rs199642525	G	GA	0.53	0.284	0.46	0.20	**1.34E-09**	0.079	0.01	0.32
15	78911181	rs8040868	C	T	0.15	0.504	0.35	0.42	**7.06E-11**	0.083	0.01	0.38
15	78915864	rs10851907	A	G	0.10	0.574	0.35	0.43	**3.97E-10**	0.079	0.01	0.39

Note: A = allele; BP = base pair; Chr = chromosome; MAF = minor allele frequency (calculated from the data); SE = standard error;

[a] In the statistical analyses of the CPD variable, original categorical observations were replaced with class means of CPD (1.5, 3.5, 8, 13, 17.5, 22.5, 32.5, and 45 cigarettes per day, respectively). Regression coefficients can therefore be interpreted as the average change in number of cigarettes smoked per day when the number of minor allele is increased by one.

[b] Continuous CPD coded as a log-transformed (natural log) value

[c] base pair position according to Build37 of the human genome

[d] rs-number for the single-nucleotide polymorphism (SNP)

[e] effect allele (minor allele)

[f] alternative allele (major allele)

[g] p-value associated with beta

[h] beta coded against the reference allele
